# Effectiveness of a Family-Caregiver Training Program in Home-Based Pediatric Palliative Care

**DOI:** 10.3390/children8030178

**Published:** 2021-02-26

**Authors:** Lourdes Chocarro González, Manuel Rigal Andrés, Julio C. de la Torre-Montero, Marta Barceló Escario, Ricardo Martino Alba

**Affiliations:** 1 Cuidados Paliativos Pediátricos, Hospital Infantil Universitario Niño Jesús, 28009 Madrid, Spain; manuelrigal@gmail.com (M.R.A.); marta_7770@yahoo.es (M.B.E.); Ricardo.martino@unir.net (R.M.A.); 2 Faculty of Health Sciences, Universidad Internacional de la Rioja (UNIR), 28040 Madrid, Spain; 3 San Juan de Dios School of Nursing and Physical Therapy, Comillas Pontifical University, 28350 Madrid, Spain; juliodelatorre@comillas.edu

**Keywords:** pediatrics, palliative care, parents, health promotion, education

## Abstract

*Background:* Pediatric palliative cares involve the whole family, along with the interdisciplinary pediatric palliative care (PPC) team. The commitment of the PPC team and the engagement of the family at different levels can play a key role in advancing a better quality of life in children and families. *Method:* A descriptive pre-post educational intervention study was carried out. The creation of a training program (with the term “school” used to denote this effort) strives to prepare caretakers to master the skills as well as provide support for the care of children with serious conditions requiring palliative through home-based initiatives. The analysis includes aspects of learning and satisfaction with the activity in a final sample of 14 families who had one child enrolled into a home-based palliative care program. *Results:* After the educational intervention in our school, the mean score of the theoretical evaluation was 9.14 points (SD 0.96), showing improvement with respect to the initial assessment, (mean diff. of +0.98 points). Although the analysis of all conceptual areas demonstrates a trend towards a positive impact of the intervention, feeding-related instruction saw the highest level of improvement, with a mean difference of +1.43 points. All enrolled parents expressed having a very positive experience during their participation in the educational program. *Conclusions:* The educational program showed a positive trend in the acquisition of knowledge and skills, resulting in a positive impact on the self-perception of their abilities. This psycho-educational space allowed them to share their experience of daily care for a child with complex needs with other families, showing them that they were not alone and that they could help each other.

## 1. Introduction

The European Association for Palliative Care for children and teenagers defines life-limiting disease as the condition in which early death is not unexpected; and life-threatening disease as that having a high probability of early death, while acknowledging also some possibility of survival (IMPACCT—European Journal of Palliative Care, 2007 vol. 14, n° 3, 109–114). The Association for Children’s Palliative Care (ACT) and the British Royal College of Pediatric and Child Health (RCPCH) describe four archetypes that help framing the evolution of a patient with any “life-limiting” disease, who could benefit from a palliative approach [1].

The expert panel in charge of developing the criteria set for palliative care for the Spanish National Public Health System estimated that between 900 and 1500 patients under the age of 20 in Spain die from life-limiting diseases every year, albeit specific studies tackling this topic are necessary. In the last 5 years under study, an average of 375 people under the age of 20 die annually in the Madrid Autonomous Region. Over half of these (55.4%) died of life-threatening diseases, as previously foreseen. We estimate that from a total of 1,300,917 children living in the Madrid Autonomous Region, between 1300 and 2100 children live with a life-threatening disease. This is the goal number of patients to be attended by a Pediatric Palliative Care Unit [2] in our region.

Home Hospitalization (HH) is an innovative, effective, and quality type of home-based care that allows patients to stay at home while receiving daily professional medical and nursing care as close as possible, in quantity and quality, as would be received within the hospital [3]. Home hospitalization aims to reduce hospital admissions, promote care continuity, and facilitate care until the end of life. It is a more humanitarian health care model that takes into consideration the preference of most of the children and their families to stay in their familiar environment [4]. Provision of enteral nutrition through nasogastric feeding tubes or gastrostomy tubes, administration of parenteral medication through subcutaneous or central line routes, and the management of tumors or complex wounds are among the many services supplied in home hospitalization programs.

Children with palliative needs are characterized by a high complexity in the management of their disease. It is necessary for caretakers to have to learn specific care to be able to handle medical technology safely. Health education emerges, therefore, as an essential element to train caretakers and provide them with the necessary tools to handle the technical aspects of their child’s care. Previously, our hospitals have created educational programs for patients with epilepsy, have required liver transplantation, or have high-risk asthma [5,6,7,8].

These parents feel great responsibility in delivering care for their child at home [9]. This hard work requires constant vigilance [10], and thrusts parents into a role where they must act as if they were real experts with inadequate training at the time of hospital discharge. While their greatest hopes are focused on managing symptoms and illness, maintaining a work/family balance, and maintaining a good quality of life are challenges. [11].

While home hospitalization strives to provide good quality of life for the children and their families, it also requires special health care education for caretakers and involves a change in the caretaker’s role as they need to carry out tasks that go beyond activities specific to the parental bond. Parents may feel overburdened and they may be conflicted about their dual role as parents and nurses [12,13,14]. In our experience we have noticed that parents find airway suctioning difficult, more so than administering oxygen therapy and feedings through a gastrotomy device [15].

Many psycho-educational programs have been developed in the field of palliative care of adults with cancer, aiming to prepare the families in palliative care both in the short and long term [16]. In the pediatric palliative field, Martínez concludes from his study in 2016 that a personalized care training for the main caretaker managing the child at home increases the perception of control and may expedite assumption of care [17].

Caretakers usually feel supported by the palliative care team, and over time they increasingly appreciate their interventions. These teams are interdisciplinary, are available 24 h, and they can ensure care continuity in the home, in the hospital and in the hospice [18].

A widely-used concept in palliative care is that of an “active patient”, who is willing to learn about his or her disease. Another concept is that of an “expert patient”, who is an active patient able to transmit his or her knowledge to others [19] with review, as necessary, to assess the skills and knowledge of these patients or families [20].

This study aims to evaluate whether an educational intervention improved caretakers’ self-competence, security, and knowledge regarding the administration of respiratory care, feedings, medication administration, hygiene, and skin care in the context of home hospitalization.

A secondary objective was to evaluate the impact of this educational intervention (the school for parents) on the number of healthcare-related telephone calls made by enrolled parents.

## 2. Materials and Methods

### 2.1. Design

We present a descriptive pilot study of an uncontrolled experimental pre-post educational intervention.

### 2.2. Population

We included a non-probabilistic sample of parents or main caretakers—defined as one who devotes more than 4 daily hours or more to direct care of their child. The following were criteria for inclusion:

- Age 0–24 years;
- Presence of a complex medical condition (as defined by Cohen et al., i.e., requiring respiratory support (home oxygen therapy, and/or respiratory suction device), and/or enteral nutritional support (nasogastric tube, gastrostomy button or tube, and/or enteral feeding pump)) [21,22,23];
- A patient of the pediatric palliative care team receiving home-based services between July and November 2018 and who had received at least two home visits from the clinical team, including the “nursing visit” for initial training in home care techniques;
- Potential participants were excluded if:
- The patient was diagnosed an oncological disease or belonged to ACT Group I, or had a life expectancy shorter that 4 months, as estimated by their care team;
- The parent was not able to speak, read, and/or write in Spanish.

A second parent was allowed to participate in the educational offering if the primary care-giving parent was already enrolled in the educational program; all parents had to commit themselves to attend at least 75% of the lessons to be included in the analysis.

**Figure 1:**
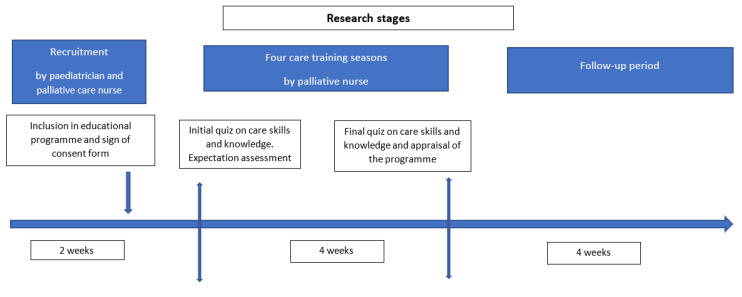
Research stages.

Subject-recruitment was performed by a pediatric palliative care (PPC) unit member (doctor or nurse), who collected data of initial assessment after the first home visit, once the child had been admitted in to the PPC program. Following evidence of compliance with inclusion criteria, their participation on the educational program was confirmed via a telephone call (Figure 1). Participants were required to read and sign an Informed Consent Form (ICF), as the project was granted the favorable report from the HUINJ Research Ethics Committee (Internal Code R-0021/18; Record 07/18 of April 24).

### 2.3. Intervention

The educational intervention was configured in training sessions, over the course of one month, with content focused on the basic needs of children with palliative needs. It was necessary to consider the potential challenges that caregivers encountered to attend the program; therefore, three different groups with different start dates were organized, with 6 participants in each group. The educational sessions took place in a conference room in Hospital Niño Jesús, which is a leading children’s hospital and a national pediatric referral center. The educational sessions were conducted by two PPC nurse specialists, one acting as trainer and the other one acting as an observer. One session was organized for each of the four thematic areas: respiratory management, feeding, hygiene and skin care, and medication administration in the home (see Table 1).

Each educational session lasted 90 min, with the first 30 min consisting of a didactic lesson on the subject by the trainer nurse, and 60 min focused on practical skills (handling care devices) and to clarify questions elicited by the group of participants. Data were entered into the study logs in the hospital, the same day, following European regulation and Spanish laws regarding data protection of special interest (children).

The contents of the didactic units were created by the members of the PPC unit, based on WHO recommendations, the Oxford Manual of Palliative Care and the Manual of Child Neurology, and adapting the language to the participants [24,25,26,27,28,29].

### 2.4. Data Collection

Three data collection forms were designed ad hoc by the investigators for this study. Table 2 shows what areas where evaluated, who was the informer and the timeline of evaluation along the educational program. Data on knowledge and skill assessment included both structured questions and open-ended comments. Structured questions were rated to a comparable scale between thematic areas, in which 0 is the lowest and 10 is the highest score. The questionnaires were prepared following the Regional Directorate of Madrid’s Methodological recommendations to prepare an educational project [23].

Regular home care visits by the PPC members continued throughout Home Hospitalization during the training period. A record of out-of-hours calls is regularly registered as part of the PPC unit home care assistance organization. Data on the number of phone calls and subject of the call by the caretakers participating in the intervention were collected prior and after the educational program. The follow-up period was variable depending on when the patient was included in Home Hospitalization program and the time of death after de intervention. Accounting for this difference in follow up, the number of calls per week was used as outcome variable.

### 2.5. Statistical Analysis

A descriptive analysis of the baseline characteristics of the patients whose caretakers were offered to participate in the training program was performed. Quantitative variables are summarized by their means and standard deviation, except the data related to the family (showed in Table 3). To identify possible selection biases, characteristics of patients whose caretakers participated in the intervention were compared against those who did not participate. Analysis of learning outcomes compares the averages of the theoretical test marks and the self-perception graded by the caretakers before and after the intervention, both overall, and on each of the conceptual areas of the program (respiratory care; feeding; hygiene and skin; and medication administration).

In the secondary aim of studying the impact on the number of care-related out-of-hours calls, the number of weekly calls was calculated in the follow-up periods before and after the intervention registered. The mean difference was calculated per week and per patient.

Software programs used for data handling included Microsoft Excel Office 365 MSO^®^ v1909 as database; and IBM SPSS Statistics^®^ (IBM Corp. Released 2015. IBM SPSS Statistics for Windows, Version 23.0. IBM Corp, Armonk, NY, USA) for their statistical data.

## 3. Results

**Figure 2:**
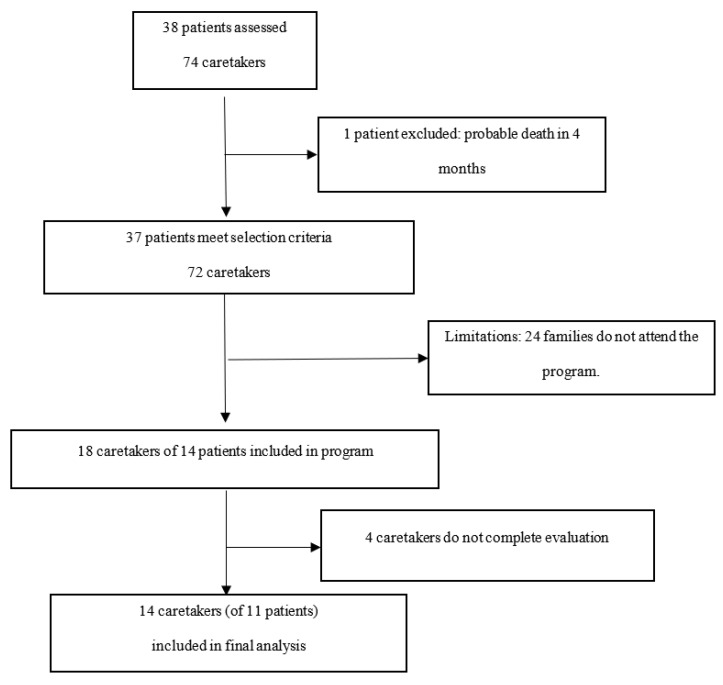
Study Flow chart. Diagram of assessed patients and caretakers participating in the program.

In the period July–November 2018 a total of 38 patients of the Hospital Infantil Universitario Niño Jesús (HUINJ) received care in their home by the PPC and considered for participation in the program as a family. Of these, one case was excluded for not meeting the selection criteria. Of this sample, 18 caretakers (all of them parents) of 14 of these patients, accepted enrollment in the study (Figure 2). When both parents acted as main caretakers, and both attended the program, both were included in the study. Only 4 parents were excluded for not completing the assessments.

Table 3 shows clinical characteristics of the patients whose caretakers accepted participation in the study as well as care complexity and need for medication of these patients.

Baseline characteristics of patients whose caretakers accepted participation in the study were analyzed and the compared to those of the 24 patients whose caretakers did not participate.

No significant differences were found in any characteristic, except in the degree of cognitive impairment, which was greater in the participant group with respect to the non-participant group (moderate or severe impairment 85.7% vs. 83.3%).

Non-participant caretakers had similar socio-demographic characteristics, many of them lacked an appropriate extra-familial support to allow them to attend programmed educational activities. Most of the 24 families that did not participate (99%) did not have anyone to look after the child to go to the school, and they related having difficulties to get to the location. One mother said she preferred leisure activities to attending a school of these characteristics. A total of 38% of them also had other additional responsibilities (they lived with a dependent adult in one case, and the rest have more young children they needed to take care of).

The social and family situation of the 14 patients whose caretakers were included in the program is summarized in Table 4. All of them identified a parent as the main caretaker, with the mother in 86% (12 out of 14) of the cases. The most frequent age group was 40–60 years (8 out of 14), and most of the enrolled parents had a university education. A language barrier was identified in one case, but it did not compromise direct communication between the parent and the healthcare professionals. In half of the cases, the main caretaker remained employed, 5 of them were unemployed, and 2 were in other, unspecified situations. Most of the parents (9 out of 14) indicated devoting more than 12 daily hours to the child’s care. 11 out of the 14 children had a recognized disability, severe (more than 65% in the WHO classification) in 8 of the cases. Most of the patients attended some type of center during the day. 5 attended an educational establishment, and 3 a specialized day-care facility; 6 of the 14 children received home care only. In most cases (12 out of 14), the families acknowledged having at least two main caretakers. 11 of them lived in a two-parent family, with a median of 1 sibling. These characteristics of patients whose parents participated in the study do not differ in the analysis of those who did not participate. When performing the comparison with caretakers who did not participate in the study, a different distribution of the education level and hours devoted to care was appreciated. The level of studies in the non-participant group was predominantly secondary education (37.5%), with university studies in only 29.2% of the cases. They also endorsed devoting more than 12 daily hours of care in a higher rate (87.5%).

Inclusion in the program was not restricted to the main caretaker. Both parents participated in 4 cases. 18 caretakers of the 14 patients described above started the program. 4 of them, however, did not complete the evaluation. Therefore, 14 parents (of 11 patients) were included in the statistical analysis (Figure 2) to assess the impact of the program, which is presented below.

Impact on knowledge and skills: Prior to the development of the training program, participants took the theoretical assessment test, with an average score of 8.15 point (over a maximum of 10) and a standard deviation of 1.36 points. Table 5 shows the mean score in the different conceptual areas (respiratory care; feeding; hygiene and skin care; and drugs) before and after the test.

After the program, the mean score of the theoretical evaluation increased to 9.14 points (SD 0.96), showing an improvement with respect to the initial assessment with a mean difference of +0.98 points. Although the analysis of all conceptual areas demonstrates a trend towards a positive impact of the educational intervention, the enteral nutrition educational intervention showed the highest impact, with a mean difference of +1.43 points.

Impact on self-competence: Participants self-assessed the level of knowledge they thought they had in each conceptual area (respiratory care; feeding; hygiene and skin care; and medication administration) on a scale of 1 (minimum score) to 10 (maximum score). Prior to the intervention, they assessed their overall capacities at a mean of 6.79 points (SD 1.28). Table 6 shows self-given scores in the 4 mentioned areas.

At the end of the program, participants improved self-perception on their own capacities in all areas. Assessment in the skills set improved to 8.50 points. Table 6 shows the subsequent scores, and the mean difference obtained.

Impact on the number of out-of-hours phone consultations: The on-call (out of hours) consultations register of the 11 patients whose caretakers participated in the program and completed evaluation was analyzed. The follow-up mean in the registry before and after the intervention for the 11 patients was 38 weeks (21–47 weeks range). At the time of analysis, the mean of follow-up after the intervention was 16 weeks (2–21 weeks range). In relation to these 11 patients, 72 on-call phone consultations were attended before the program, and 81 consultation afterwards. The rate of consultations per week per patient was used to compare both periods (Table 7).

Before the intervention, the mean of out-of-hours telephone calls for any cause was 0.18 consultations per week and patient. In the follow-up after the intervention, the mean of calls for any cause increased to 0.69 consultations/week-patient, which means 3.8 times more consultations. This showed an increase, with a mean difference of 0.51 calls/week-patient. In the analysis of consultations by reason for the call (Table 7), an increase in calls regarding symptoms was observed (mean difference 0.44 consults/week-patient). Calls for this reason explain most of the phone calls: they were 79.1% of the total received before the intervention, and 82.7% after it. No differences in calls for other reasons were observed (queries on medication, devices, administration, and others) between the periods before and after the intervention.

Participants’ lived experience and satisfaction with the educational program: All participants expressed having a very positive experience with their participation in the educational program. They point out a few aspects: (1) they had the chance to share with other parents their experience of providing care for the child at home and especially how they solved everyday difficulties, e.g., going out for a walk with their child while on home oxygen, or needing a suction machine, or having a gastrostomy tube, thus normalizing their everyday life; (2) they had the chance to see that they are not alone, and that they could help each other out; and (3) they gained confidence in reporting via telephone what was happening to their child, since they acquired more specific technical terminology.

## 4. Discussion

These children had complex care needs [30,31,32], although their parents had been administering care in their home, there may be a need to improve communication with the palliative care team and to refresh skills and knowledge which may have changed over time [20].

All caretakers participating in the educational program devoted over 12 daily hours to their child with complex care needs. Most of them were mothers. These caretakers reflect the “active patient” pattern [19], as they show a good disposition to not only cover their child’s complex needs, but also the desire to improve care provision. Although they perform care on a daily basis, over time they may deviate from the proper technique, so health education becomes an indispensable main axis for care of these children to be carried out in their home [20].

The educational intervention presented in this study shows a modest positive impact on the knowledge and skills these caretakers need to provide care for their child. Notably, their knowledge and skills were considerably high (average 8.15 over 10) at the time of the initial evaluation before the intervention, so the room for improvement was small. It is interesting, however, to highlight that the intervention showed a more remarkable positive effect when evaluating their self-perception of skills and capabilities. These findings may suggest that these caretakers perceived themselves as less competent than what the theoretical knowledge test denoted. This self-perception improved at the end of the educational program. The log of out-of-hours telephone calls performed by these caretakers after the educational intervention increased in number (consultation about symptoms). During the sessions, a positive atmosphere of trust between caretakers was created, to a point where in one of the groups some caretakers who were reluctant to have a gastrostomy performed child, subsequently agreed to the intervention.

In any case, in the pediatric palliative care field, the patient’s family and caretakers are active patients who are motivated to learn to provide better care for their child. In some cases, they are “expert” caretakers who could, in turn, instruct other parents/main caretakers in the same care complexity situation. In pediatrics, we take the child and their family as the care unit, which is why we consider caretakers to be active “patients”. What we mean by “active”, is that they show a good disposition to acquiring knowledge, skills, and self-confidence in order to manage their child’s health [19,33].

Proactive palliative care interventions, and empathic communication with caregivers can improve care [34]. Our educational intervention have improved how caregivers express children’s symptoms.

Phone consultations are of very frequent use, even more in pediatrics, and they give a high level of satisfaction to the families [35,36]. One expectation of this work was a decrease in phone consultations. However, their number did not decrease. This may be because of the child’s worsening condition due to the natural history of the disease, or because they have more knowledge, giving rise to more questions; or because the self-perception they learned is making them more comfortable and confident, allowing them to “dare” to do more things for which they need the professional’s confirmation.

We want to highlight the importance of setting up a school, using dedicated space and appropriate equipment if possible—especially when the degree of technological support is high, e.g., when home ventilator support is contemplated—for caretakers in the context of pediatric palliative care. The small sample shows the delicate situation that these families experience. We believe that internal validity is robust, and although we understand that it is difficult to make a generalization, the aim of this work is to point out that there are significant advantages in palliative care education, and the need for the application of elements of instruction in this area.

The program is perceived as a psycho-educational space, of mutual trust, where peer learning is fundamental, as it stimulates self-confidence and peer perception of usefulness. Future research lines could include larger samples and use phenomenological design to explore the subjective experience of actors. The course structure improves perception, and peer learning increases self-confidence. Furthermore, the plasticity of the care team to adapt to the environment, with heterogeneous socioeconomic conditions where there was a good interaction, was appreciated.

When this study was conducted, we were not in a pandemic situation, but COVID-19 has increased the social isolation of these families. On the other hand, the pandemic has allowed us to recognize the value of new communication technologies to get closer to these families through telemedicine modalities. We believe that new online editions of the educational program will allow families who were unable to do so in person [37].

## 5. Limitations

The main limitation of the paper is the small sample size, in large part, due to lack of family support needed to allow parents to access this program. This uncontrolled experimental study deals with an interesting and unexplored topic: structured education of caregivers of a child with a life-limiting condition, and as such it can potentially make valuable contributions to the field. Bearing in mind the limitation of the sample, we consider that the importance of showing very positive results is relevant for professionals and patients, as well as for their families. Other limitations of this work were found in the lack of class attendance from a large group of caretakers who, due to justified reasons, could not actively participate as they would have liked. The main reasons were economic, seeming that caretakers who had less external support were more likely to refuse participation. Pediatric palliative care poses a challenge to the care system, especially when located outside the hospital complex. In our case, knowing that the effect of the intervention on knowledge and skills seems important we think more work is required with a wider number of participants to confirm this.

Another limitation of the study, related to the analysis of the intervention’s influence on the frequency of out-of-hour calls, is that it does not take into account the influence of the change in the children’s clinical situation.

## 6. Conclusions

Caretakers of children with life limiting conditions show high skills and knowledge in the tasks that are needed to develop their role. An educational program may strengthen these capabilities and improve levels of confidence. This study shows that the provided intervention had a particularly positive effect in caretakers’ self-perception and confidence about the outstanding caring skills they already had.

Although the number of out-of-hours telephone calls from the caregivers to the PPC team after the intervention did not decrease, they were more focused on the description of symptoms. A high level of commitment of the caregivers was found.

In summary, psycho-educational space emerged in our school for parents of children with complex health care needs in the setting of home-based palliative care that allowed them to share their experience of daily care for their children. In addition, they have seen that they are not alone and that they can help each other.

## 7. Distinctive Contribution of This Work

A large body of evidence already exists on educational programs focusing on assessing the satisfaction of subjects involved. Our study provides assessment on clinical impact, summarized in improvement in knowledge, capacities, skills and in the description of clinical signs in their sick children, acquired in the training sessions.

Likewise, the importance of the creation of a psycho-educational space that allowed caretakers to share experiences in daily home care for their sick children is identified. Health education not only impacts the content taught and the skills acquired, but also safety and self-confidence.

The Transparent Reporting of Evaluations with Nonrandomized Designs (TREND) statement was followed with the intention of being able to present to readers a robust methodology in difficult working and research conditions [38].

Future research lines: In the future, we will continue our work with the school for parents. We will also create an online course due to the Covid pandemic, and we will further investigate regarding the participant’s experience of actors.

## Figures and Tables

**Table 1:** Thematic areas.

Subjects	Contents
Respiratory	Anatomy and physiology; assessment of breathing patterns; respiratory infections; handling of breathing devices
Feeding	Anatomy and physiology; dysphagia; nutritional and dietary advice; handling of feeding devices
Hygiene and skin care	Posture changes; hygiene; wellbeing; maintaining an adequate posture
Medication	Groups of drugs; administration routes; home medicine chest

**Table 2:** Assessment instruments.

Assessment Instruments			
Assessment Tool	Description	Informer	Scheduled for Assessment
Self-assessment of session attendees. Question example: Quantify (between 1 and 10) the level of knowledge you think you have about breathing care.	Each participant quantifies their own level of knowledge before and after attending the course	Participant	Before the first session and at the end of fourth session
Theoretical test	The test includes five multiple choice questions for each lecture. Pre post grading of knowledge quiz to assess initial and final knowledge in each topic.	Participant	Before the first session and at the end of fourth session
Participants’ experience or satisfaction with the sessions.	Open-ended questions that each participant anonymously responded in writing at the end of the last lecture. “What did you think of these sessions?” “Do you think they will be of help in your everyday administration of care for you child?” “Which skill or care do you find hardest to perform?”	Participant	At the end of fourth session

**Table 3:** Characteristics of the patients whose caretakers participated in the program.

Sample (*n* = 14)			
Childs age	9.6	Follow-up time in home care program	13.3
(average, years)	(SD = 7.1)	Deceased after 6 months from intervention	7
**Gender**		**Pathology**	
Female	6	Tetraparetic Cerebral Palsy	5
Male	8	Congenital Malformation Syndrome	1
		Epileptic Encephalopathy	4
		Hallenvorder-Spatz	1
		Mucopolysaccharidosis	1
		Neurofibromatosis	1
		Congenital Metabolic Disorder	1
**Cognitive impairment**		**Verbal Ability**	
None or minor	1	Yes	1
Moderate or severe	13	No	13
**Sensory Deficit**		**Mobility**	
None	5	Wanders	1
Visual	1	Bed-Sofa	5
Hearing and visual	6	Wheelchair	5
		Bed-ridden	2
**Care and Devices**		**Medication**	12
Nasogastric tube	4	Anti-epileptic drugs	8
Gastrostomy tube	8	Muscle relaxants	2
Tracheostomy	2	Neuroleptic drugs	9
Invasive ventilation	1	Bronchodilators	1
Non-invasive ventilation	4	Opioids	7
Domiciliary oxygen	14	Minor analgesics	5
Airway secretion aspirator	11	Analgesic adjuvants	4
Subcutaneous catheter	0	Anxiolytics/sedatives	5
IV line	1	Antacids/Laxatives	8
Diapers	12		

**Table 4:** Sociodemographic characteristics of the parents participating in the program (main caretakers) and family organization around child’s care.

Sample (*n* = 14)			
**Gender**		**Level of studies**	
Female	12	Primary	2
Male	2	Secondary	3
		University	9
**Age**		**Language Barrier**	
16–24 years	1	No	13
24–40 years	4	Yes, direct communication.	1
40–60 years	8	Yes, needs translator	0
>60 years	0		
**Work situation**		**Daily hours dedicated to care.**	
Employed	7	2–4 h	2
Unemployed	5	4–8 h	1
Others	2	8–12 h	0
		>12 h	9
**Child using daytime resources.**		**Receives benefits from Dependency ACT**	
In school	5	Yes	7
Daycare social-sanitary centre	3	No	3
Home only	6	DK/DA *	4
**Family unit**		**Routine caretakers at home**	
Two-parent	11	Only one	2
Single parent	3	Two	10
Home cohabitants	3 (3–4)	Three or more	2
Number if siblings			
(median and quartiles)	1 (0–2)		

* DK/DA: Doesn’t know / doesn’t answer.

**Table 5:** Mean score in the theoretical knowledge test before and after the training program, and the mean differences.

	BEFORE Mean (SD)	AFTER Mean (SD)	Mean Difference
**Respiratory Care**	6.90 (3.05)	8.10 (2.52)	1.19
**Feeding**	8.57 (2.34)	10.0 (0)	1.43
**Hygiene and skin**	7.86 (2.48)	8.81 (1.66)	0.95
**Medication Administration**	8.29 (1.82)	9.64 (1.34)	0.36
**Average**	8.15 (1.36)	9.14 (0.96)	0.98

**Table 6:** Mean score in self-perception of knowledge before and after the training program, and the mean differences.

	BEFORE Mean (DS)	AFTER Mean (DS)	Mean Difference
**Breathing**	6.29 (2.40)	8.00 (1.18)	1.71
**Feeding**	6.64 (1.86)	8.5 (1.16)	1.86
**Hygiene and skin**	6.64 (1.28)	8.64 (0.63)	2.00
**Medication**	7.57 (1.16)	8.86 (0.95)	1.29
**Average**	6.79 (1.28)	8.50 (0.81)	1.71

**Table 7:** Mean number of consultations in the periods before and after the training program. Expressed as consults per week per patient.

	BEFORE Mean (DS)	AFTER Mean (DS)	Mean Difference
**Symptoms**	0.14 (0.09)	0.59 (0.74)	0.44
**Questions about medications**	0.02 (0.04)	0.02 (0.04)	0.00
**Devices**	0.01 (0.01)	0.06 (0.14)	0.05
**Administrative and other**	0.01 (0.02)	0.02 (0.05)	0.01
**Average**	0.18 (0.11)	0.69 (0.79)	0.51

## Data Availability

The data collected for the study has been stored in a dissociated database accessible only by the research team. The study complies the Spanish Ley Organica 3/2018 for Protection of Personal Data, and the European Regulation (RGPD 2017/679).
